# Identification and Characterization of a Novel Chemotype MEK Inhibitor Able to Alter the Phosphorylation State of MEK1/2

**DOI:** 10.18632/oncotarget.747

**Published:** 2012-12-01

**Authors:** Takayuki Yoshida, Junya Kakegawa, Takayuki Yamaguchi, Yoshiji Hantani, Nobuyuki Okajima, Toshiyuki Sakai, Yoshihiro Watanabe, Motonao Nakamura

**Affiliations:** ^1^ Pharmaceutical Frontier Research Laboratories, Central Pharmaceutical Institute, Japan Tobacco, Kanazawa-ku, Yokohama, Kanagawa, Japan; ^2^ Biology and Pharmacology Research Laboratories, Central Pharmaceutical Research Institute, Japan Tobacco, Takatsuki, Osaka, Japan; ^3^ Chemistry Research Laboratories, Central Pharmaceutical Research Institute, Japan Tobacco, Takatsuki, Osaka, Japan; ^4^ Department of Molecular-Targeting Cancer Prevention, Graduate School of Medical Science, Kyoto Prefectural University of Medicine, Kawaramachi-Hirokoji, Kamigyo-ku, Kyoto, Japan

**Keywords:** JTP-74057/GSK1120212/trametinib, chemical probe-affinity chromatography, novel MEK inhibitor, unphosphorylated MEK (u-MEK)

## Abstract

A small molecule compound, JTP-74057/GSK1120212/trametinib, had been discovered as a very potent antiproliferative agent able to induce the accumulation of CDK inhibitor p15^INK4b^. To conduct its drug development rationally as an anticancer agent, molecular targets of this compound were identified as MEK1/2 using compound-affinity chromatography. It was shown that JTP-74057 directly bound to MEK1 and MEK2 and allosterically inhibited their kinase activities, and that its inhibitory characteristics were similar to those of the known and different chemotype of MEK inhibitors PD0325901 and U0126. It was further shown that JTP-74057 induced rapid and sustained dephosphorylation of phosphorylated MEK in HT-29 colon and other cancer cell lines, while this decrease in phosphorylated MEK was not observed in PD0325901-treated cancer cells. Physicochemical analyses revealed that JTP-74057 preferentially binds to unphosphorylated MEK (u-MEK) in unique characteristics of both high affinity based on extremely low dissociation rates and ability stabilizing u-MEK with high thermal shift, which were markedly different from PD0325901. These findings indicate that JTP-74057 is a novel MEK inhibitor able to sustain MEK to be an unphosphorylated form resulting in pronounced suppression of the downstream signaling pathways involved in cellular proliferation.

## INTRODUCTION

Cancer cells utilize surface receptors for growth factors and signaling molecules of the Ras–Raf–MEK–ERK pathway to promote cell growth, and this pathway is critical of outgrowth even in Ras/Raf-unmutated cancer cells [1-3]. Inhibitors of growth factor receptor EGFR and Raf kinase suppress the activation of downstream kinases (i.e., MEK and ERK) in this signaling pathway, thereby offering significant benefits for many cancer patients [4-8]. At the same time, these inhibitors have clarified the existence of insensitive cancer types and the emergence of resistant mechanisms against them [9-12].

MEK1 and MEK2 are kinases that link the cascade between Raf and ERK, and their inactive unphosphorylated forms (u-MEK1/2) become phosphorylated (p-MEK1/2) and hence activated by Raf kinase. Since MEK plays critical roles in tumor growth and progression, some MEK inhibitors have been developed and evaluated as potential therapeutic agents [13-16]. However, these drugs did not show significant benefits for cancer patients, and have drawbacks including adverse events caused by off-target binding, limited potency, and an excessive degree of chemical similarity that precludes mechanistic differentiation [17-19]. To overcome these limitations, it has been clear that new MEK inhibitors possessing different chemical and pharmacological characteristics are needed.

In general, cell phenotypic assays offer a feasible method to identify novel drugs with good potency and efficacy *in vivo*. JTP-74057 was originally discovered as a CDK inhibitor p15^INK4b^-inducing agent in various cancer cells, based on that p15^INK4b^ possesses inhibitory activity of cell cycle progression and usually retains as an unmutated form even in cancer cells [20]. Screening of our chemical library followed by extensive medicinal chemistry enabled us to identify JTP-74057 as a promising candidate compound. It has been revealed that JTP-74057 is a very potent antitumor agent *in vitro* and *in vivo* [21, 22]. Importantly, this compound exhibited over 50-fold selectivity for cancer cells relative to normal tissues and hematopoietic cells, suggesting that its molecular targets and mechanism of action could heighten our understanding of cancer cell growth and aid the development of novel anticancer agents. Actually, JTP-74057/GSK1120212/trametinib has recently been demonstrated to be a first-in-MEK inhibitor able to improve the progression-free survival of BRAF-mutated advanced melanoma patients with its ideal pharmacological and pharmacokinetic profile [23, 24].

We describe here that molecular targets of this compound are MEK1 and 2, and that JTP-74057 possesses novel characteristics partly different from previously known MEK inhibitors. Namely, drug-affinity chromatography using chemical probes identified MEK1/2 as directly binding molecules, and JTP-74057 showed an allosteric type of MEK inhibition similar with PD0325901. Unlike PD0325901, however, it has been demonstrated that JTP-74057 shifts the MEK phosphorylation status from p-MEK toward u-MEK in several cancer cell lines and binds to u-MEK with a very low dissociation rate. In this paper, we further describe how this characteristic of JTP-74057 is relevant to its very potent and prolonged inhibition of Raf-MEK-ERK signaling in cancer cells.

## RESULTS

### Identification of MEK1/2 as molecular targets of JTP-74057

**Figure 1:**
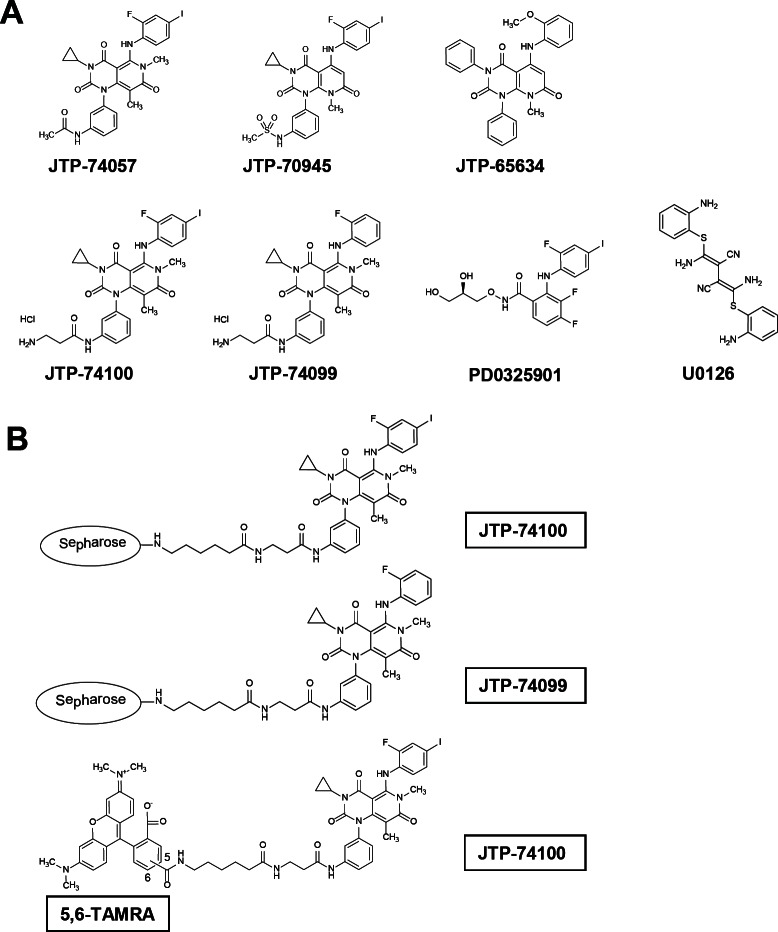
Chemical structures of JTP-74057 chemotype compounds, known MEK inhibitors and chemical affinity probes (A) The chemical structures of active compounds (JTP-74057 and JTP-70945), a minimally active compound (JTP-65634), linker derivatives used as chemical probes (JTP-74099 and JTP-74100) and known allosteric MEK inhibitors (PD0325901 and U0126) are shown. The growth inhibitory activities of each compound were as follows: JTP-74057, 0.57 nM; pentanoyl JTP-74100, 2.1 nM; JTP-70945, 0.39 nM; pentanoyl JTP-74099, 840 nM; JTP-65634, >10 μM; PD0325901, 3.4 nM. (B) JTP-74100 and JTP-74099 were conjugated with Sepharose 4B for use in chemical affinity chromatography, and JTP-74100 was linked with the 5,6-linker TAMRA for use in analyses by fluorescence microscopy and fluorescence correlation spectroscopy.

A chemical affinity method was used to identify a molecular target of JTP-74057 and other compounds from the same chemotype. Linker-conjugated compounds derived from the JTP-74057 chemotype were synthesized and their growth-inhibitory effects were examined (Figure 1A and Supplemental information). The experiments revealed that attachment of conjugation linkers only to the aniline nitrogen atom of this chemotype did not reduce their antiproliferative effects on cancer cells. Since the alkyl linker-conjugated compounds JTP-74100 (IC_50_: 2.1 nM in pentanoyl JTP-74100) and JTP-74099 (IC_50_: 840 nM in pentanoyl JTP-74099) retained their antiproliferative activities, both compounds were considered for use as chemical probes with which to prepare compound-conjugated affinity resins and fluorescence-conjugated compounds.

**Figure 2:**
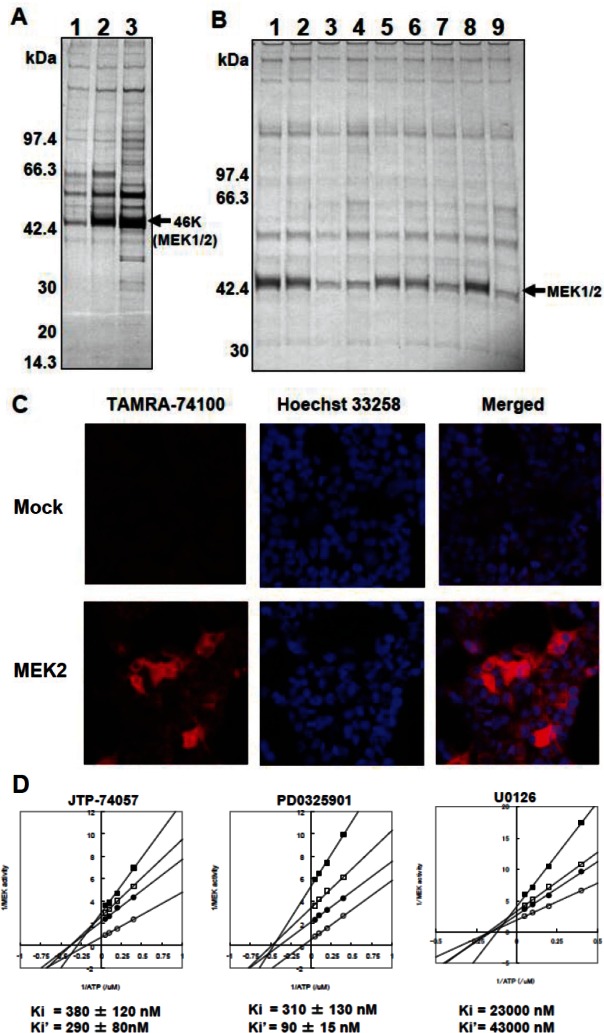
MEK1/2 as direct target molecules binding to JTP-74057 chemotype compounds (A) Affinity chromatography using compound-immobilized resins. The unconjugated resin (1) and resins conjugated with JTP-74099 (2) or JTP-74100 (3) were incubated with cytosolic proteins extracted from HT-29 cells. The pull-down samples were separated by SDS-PAGE. A 46-kDa protein bound extensively to the conjugated resin was identified by LC-MS/MS to be human MEK1 and MEK2. (B) Competitive analysis with various JTP-74057 chemotype compounds and other MEK inhibitors. The cytosolic fractions of HT-29 cells were preincubated with DMSO alone (1), 1 μM JTP-70945 (2), 10 μM JTP-70945 (3), 100 μM JTP-70945 (4), 1 μM PD0325901 (5), 10 μM PD0325901 (6), 100 μM PD0325901 (7), 100 μM JTP-65634 (8) or 100 μM U0126 (9), and then pulled down using the JTP-74100-conjugated resin. The samples were assessed by SDS-PAGE analysis to detect a 46-kDa protein band. (C) Subcellular localization of a fluorescent compound of the JTP-74057 chemotype in cells. HEK293T cells were transfected with a mock vector (upper panels) or human MEK2 cDNA (lower panels). The cells were treated overnight with 1 μM of TAMRA-conjugated JTP-74100, fixed with 4% formaldehyde, stained with Höechst 33258, and observed by fluorescence microscopy. (D) Kinetic analysis of JTP-74057 and known MEK inhibitors in the MEK–ERK enzymatic reaction. Double-reciprocal analyses of MEK enzymatic activity as a function of the ATP concentration were conducted at several fixed concentrations of the compounds. JTP-74057 and PD0325901 were set at concentrations of 0 (open circles), 0.125 (filled circles), 0.25 (open squares) or 0.5 (filled squares) μM. In the case of U0126, the inhibitor concentrations were 0 (open circles), 10 (filled circles), 20 (open squares) and 40 (filled squares) μM. The Ki (MEK *vs.* compound) and Ki’ (MEK/ATP *vs.* compound) values were calculated from the plots. The data shown are the means of two or three independent experiments.

To identify specific binding targets, we prepared three chemical affinity resins. The first was unconjugated and used as a negative control, the second was conjugated with JTP-74099 and the third was conjugated with the more potent compound, JTP-74100 (Figure 1B). HT-29 cell lysates were incubated with the individual resins and the bound proteins were extracted by pull-down assays. Figure 2A shows the electrophoresis data of these pull-down samples. Specific binding proteins, including a dominant 46-kDa protein, accumulated in the compound-conjugated resins, most significantly in the resin conjugated with the potent JTP-74100, while the unconjugated resin only bound proteins nonspecifically. The bound proteins were subjected to LC-MS/MS analysis (Supplemental information), which revealed that MEK1 and MEK2 were the major proteins bound to JTP-74100, with less extensive binding to JTP-74099 and negligible binding to the negative control resin.

To confirm that the resin conjugated with JTP-74100 specifically binds to MEK1/2, we performed competitive analyses using active (JTP-70945) and minimally active (JTP-65634) compounds of the same chemotype, in addition to PD0325901 and U0126 as known MEK inhibitors originating from different chemotypes [4, 25]. As shown in Figure 2B, JTP-70945 (at concentrations above 10 μM) clearly competed with JTP-74100-immobilized resin for binding to MEK, while the minimally active compound JTP-65634 did not compete, even at a concentration of 100 μM. The other chemotype MEK inhibitors, PD0325901 and U0126, needed a concentration of 100 μM to clearly compete with the binding of MEK to JTP-74100-immobilized resin.

Next, we determined whether JTP-74100 binds to MEK in intact cells by detecting fluorescent signals derived from TAMRA-conjugated JTP-74100 (Figure 1B) in human MEK2-transfected HEK293T cells. As shown in the lower panels of Fig. 2C, red fluorescent signals corresponding to TAMRA-conjugated JTP-74100 were distributed throughout the cytosol, but no signals were detected in the nucleus. In contrast, we detected only weak signals in mock-transfected cells. The distribution of the fluorescent signals in the MEK-transfected cells was consistent with the cytosolic localization of MEK proteins, as reported previously [26]. Taken together, these findings indicate that active compounds of the JTP-74057 chemotype directly bind to MEK proteins.

### Effects of JTP-74057 on enzyme activity

To address the effects of JTP-74057 on MEK kinase activity, we examined its ability to inhibit various enzymatic reactions of Raf, MEK and ERK, relative to that of PD0325901. The results obtained from the three kinds of kinase assays are summarized in Table 1, in addition to their antiproliferative activities toward HT-29 cells.

**Table 1:** Inhibitory activity of JTP-74057 against MEK activity Kinase assays were performed as described in the Materials and Methods. The growth inhibitory activities (IC50) of JTP-74057 and PD0325901 in HT-29 cells were measured by [^3^H]-TdR incorporation during the last 6 h of a 3-day culture period. The data shown are the means of three independent experiments.

Kinase assay	Kinase	Substrate	IC50 value (nM)	
			JTP-74057	PD0325901
MEK/ERK	p-MEK1	ERK2	290	110
	p-MEK2	ERK2	190	140
Raf/MEK/ERK	B-Raf/u-MEK1	ERK2	3.5	4.1
	B-Raf/u-MEK2	ERK2	5.3	3.6
	c-Raf/u-MEK1	ERK2	11	24
	c-Raf/u-MEK2	ERK2	7.9	6.0
Raf/MBP	B-Raf	MBP	>10,000	>10,000
	c-Raf	MBP	>10,000	>10,000
Cell proliferation (HT-29)			0.57	3.4

First, we examined the MEK–ERK kinase reaction, which was inhibited by JTP-74057 with an IC_50_ value in the sub-micromolar range, similar to that of PD0325901. Second, the inhibitory activities of JTP-74057 on the Raf–MEK–ERK kinase cascade were examined. This experiment was based on active Raf (mutated B-Raf (V600E) or truncated c-Raf), u-MEK1/2 and u-ERK2. JTP-74057 and PD0325901 strongly inhibited the Raf–MEK–ERK cascade with nanomolar potency, regardless of the isotypes of Raf or MEK used, while PD0325901 showed similar inhibitory activity. Third, we examined whether JTP-74057 inhibits the kinase activity of Raf itself. The phosphorylation of the Raf kinase substrate MBP was not inhibited by JTP-74057 or PD0325901 at concentrations below 10 μM. Under the same conditions, the recognized Raf inhibitor BAY 43-9006 inhibited Raf kinase activity at a concentration of 0.1 μM (data not shown). These findings suggest that JTP-74057 is a potent MEK inhibitor that acts *via* a similar mechanism to that of PD0325901. We also determined that JTP-74057 was around 10-fold more potent at inhibiting cellular proliferation than it was at suppressing the activity in the Raf–MEK–ERK cascade assay (Table 1).

PD0325901 and U0126 are known to be ATP-noncompetitive allosteric MEK inhibitors [25, 27]. We analyzed the kinetic properties of MEK inhibition by JTP-74057 using active p-MEK1 and inactive u-ERK2 to establish double-reciprocal plots of MEK enzymatic activity at the indicated concentrations of these compounds with ATP. As shown in Figure 2D, these plots revealed similar patterns for the three compounds, and the lines did not cross the *y*-axis. The Ki and Ki’ values represent the dissociation constants of the inhibitor from p-MEK and the p-MEK–ATP complex, respectively. These values indicated that JTP-74057 bound to p-MEK and the p-MEK–ATP complex, and that the dissociation constants of JTP-74057 were similar to or slightly weaker than those of PD0325901. These findings indicate that JTP-74057 is an ATP-noncompetitive allosteric MEK inhibitor, similar to PD0325901 and U0126.

### Alteration of MEK phosphorylation status by JTP-74057

**Figure 3:**
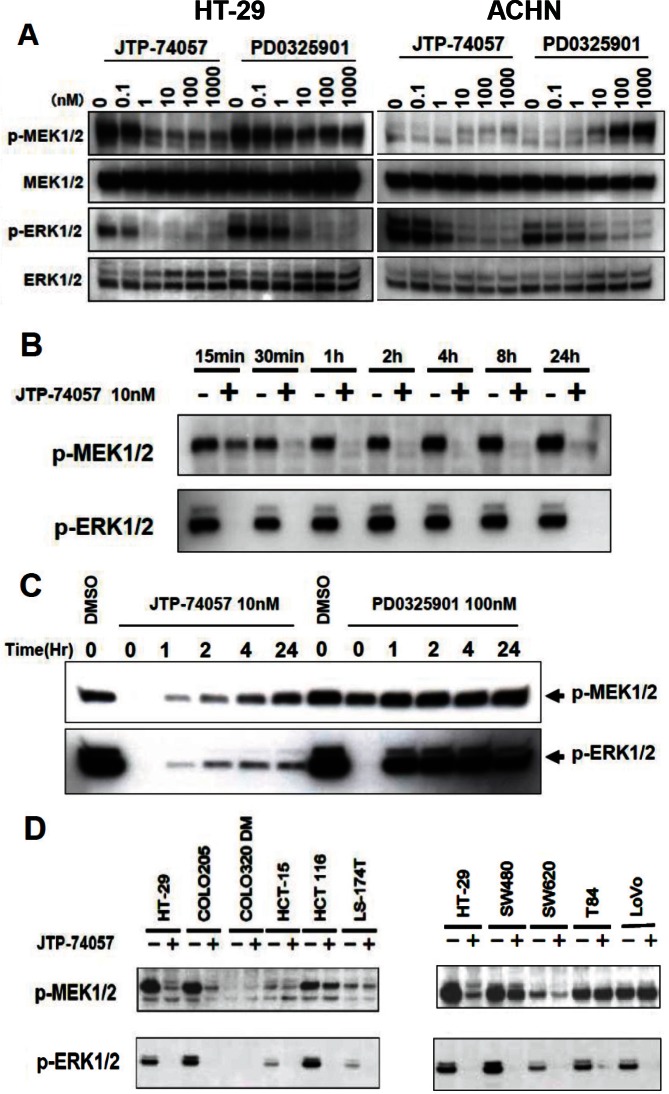
Rapid shift to and sustained state of u-MEK form by JTP-74057, but not PD0325901 (A) HT-29 cells (left) and ACHN cells (right) were treated with the indicated concentrations of each compound for 24 h, and the cell lysates were analyzed by western blotting using antibodies specific to phosphorylated and total MEK1/2 and ERK1/2. (B) Time-course analysis of the phosphorylation states of MEK and ERK in HT-29 cells treated with JTP-74057. HT-29 cells were treated with 10 nM JTP-74057 for the indicated times, and the cell lysates were analyzed by western blotting using antibodies specific to MEK or ERK. (C) Prolonged inhibition of ERK phosphorylation via sustained u-MEK status by pulsed JTP-74057, but not PD0325901. HT-29 cells were treated with 10 nM JTP-74057 or 100 nM PD0325901 for 2 h. After wash twice with PBS, the medium was replaced with inhibitor-free media and the cells were cultured for the indicated times. The cell lysates were analyzed by western blotting in terms of the phosphorylation state of MEK and ERK. (D) Alteration of the phosphorylation states of MEK and ERK by JTP74057 in various cancer cells. Individual cancer cell lines were treated for 2 h with 100 nM JTP-74057, except for the HT-29 line that was treated with 10 nM. Lysates of the cells were analyzed by western blotting using specific mAbs. The data showed that ERK phosphorylation was efficiently inhibited in almost all cell lines, except for COLO-320DM, and that the p-MEK levels decreased in HT-29, COLO-205, HCT-116, SW480 and SW620 cells, but did not decrease or decrease to a lesser extent in HCT-15, LS-174T, T84 and LoVo cells.

Next, we analyzed the phosphoprylation status of ERK and MEK to expect inhibition of downstream signaling by JTP-74057. As shown in Figure 3A, JTP-74057 and PD0325901 inhibited ERK phosphorylation, while JTP-74057, but not PD0325901, decreased the p-MEK level. It was also confirmed that inhibition of ERK phosphorylation resulted in downstream signaling to be inhibited and accumulation of p15^INK4b^ and p27^KIP1^ (Supplemental Figure 1). The inhibitory potency of JTP-74057 on ERK phosphorylation and downstream signaling was about 10-fold higher than that of PD0325901, which was consistent with its growth inhibitory activity toward HT-29 cells. These findings indicate that JTP-74057 and PD0325901 suppress ERK phosphorylation and hence affect the subsequent downstream signaling cascade, thereby inhibiting to cell cycle progression. Of particular interest is that JTP-74057, but not PD0325901, shifted the MEK phosphorylation status from p-MEK to u-MEK. In ACHN cells, since the p-MEK level was low to detect clearly, the significant decrease of p-MEK was not observed in JTP-74057 at the concentrations showing inhibition of ERK phosphorylation. At higher concentrations of PD0325901 but not JTP-74057, it was clearly demonstrated that significant accumulation of p-MEK was observed, which is often observed in cancer cells treated by conventional MEK inhibitors [28, 29].

We further determined how JTP-74057 shifts p-MEK to u-MEK in HT-29 cells. As shown in Figure 3B, the time-course analyses revealed that the level of p-MEK was decreased within 15 min and almost ablated at 30 min. To identify whether the disappearance of p-MEK was reversible, the cells were exposed to JTP-74057 for a brief period (2 h), washed and cultured in normal medium for the indicated times before measurement of the MEK phosphorylation status. As shown in Figure 3C, the disappearance of p-MEK was sustained for a considerable period after removal of JTP-74057, and the p-MEK levels gradually recovered during 24 h but were still lower than the untreated level. The level of p-ERK was dependent on p-MEK recovery, indicating that the modulation of p-MEK by JTP-74057 had downstream effects on the activation of ERK in HT-29 cells. In marked contrast, PD0325901 did not affect the MEK phosphorylation status, and the p-ERK level returned to nearly normal level within 1 h after its removal from the culture media.

In order to assess whether this shift to u-MEK by JTP-74057 is widely observed in various cancer cells, the phosphorylation status of MEK was examined in 10 cancer cell lines. As shown in Figure 3D, JTP-74057 decreased p-MEK abundance in approximately half of the cancer cell lines examined, though the decreased levels of p-MEK varied among individual cancer cell lines. These findings indicate that the JTP-74057-induced shift of MEK from p-MEK to u-MEK is not a universal phenomenon, but does occur in a wide range of cancer cell lines. These findings demonstrate a novel characteristic of JTP-74057, in that it is able to induce a rapid and sustained shift from p-MEK toward u-MEK. Moreover, the complex formed between JTP-74057 and u-MEK is resistant to phosphorylation by upstream kinases in cancer cells, thereby inhibiting ERK phosphorylation effectively.

### Unique binding mode of JTP-74057 to u-MEK

**Figure 4:**
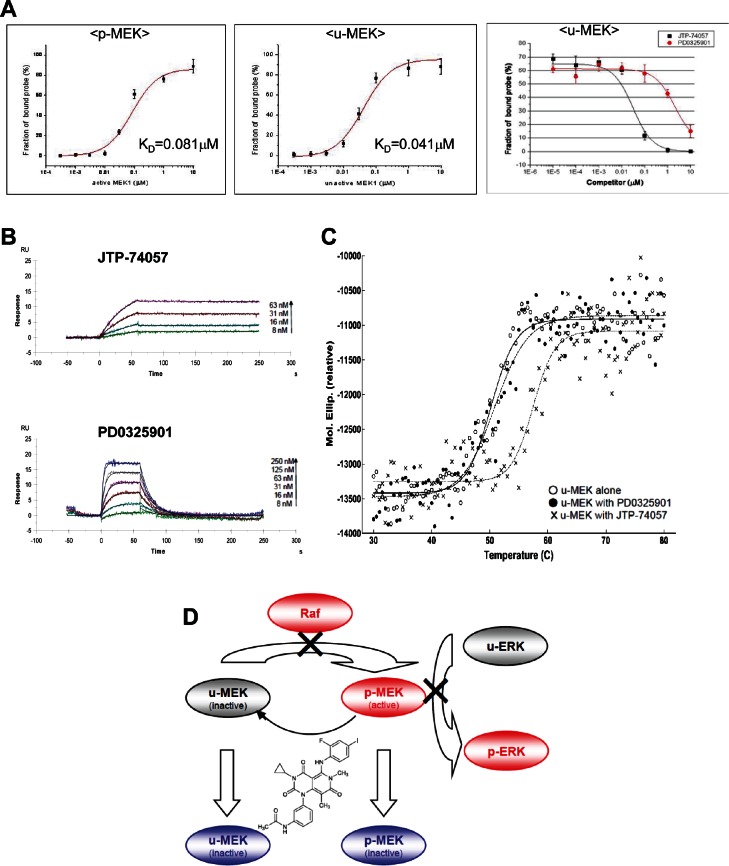
Physicochemical analyses of JTP-74057 chemotype compounds binding to MEK, and a scheme showing a novel type of allosteric MEK inhibitor JTP-74057/GSK1120212/trametinib (A) FCS analysis. p-MEK1 (left) and u-MEK1 (center) were incubated with 1.5 nM TAMRA-conjugated JTP-74100 for 30 min at 25°C. The FCS measurements were performed with a single molecule fluorescence analyzer (MF20). The calculated K_D_ values of TAMRA-conjugated JTP-74100 for p-MEK and u-MEK1 are 4.1×10^−8^ M and 2.1×10^−8^ M, respectively. In the right panel, u-MEK1 (0.1 μM) was incubated with 1.5 nM TAMRA-conjugated JTP-74057 plus various concentrations of JTP-74057 or PD0325901 to displace the reporter probe. (B) SPR analysis. After immobilization of u-MEK to CM5 sensor chips, JTP-74057 and PD0325901 were injected at the indicated concentrations. A 1:1 binding model with mass transfer was fitted to the sensorgrams. Theoretical curves (black) were overlaid on the experimental traces. The K_D_ (M) and k_off_ (s^−1^) to u-MEK are 3.5×10^−10^ and 1.2×10^−4^ for JTP-74057, and 6.1×10^−8^ and 1.1×10^−1^ for PD0325901, respectively. (C) Temperature-dependent CD analysis. Ellipticities of inactive u-MEK were analyzed at the temperature between 30 to 80 °C in the absence or presence of 10μM of either JTP-74057 or PD0325901. The *Tm* values were calculated by individual fitting curve to 50.2 °C for u-MEK alone, 50.9 °C for u-MEK with PD0325901, and 57.5 °C for u-MEK with JTP-74057. (D) A scheme showing a novel type of allosteric MEK inhibitor JTP-74057/GSK1120212/trametinib. JTP-74057/GSK1120212/trametinib binding to MEK inhibits not only the p-MEK activity able to induce ERK phosphorylation, but also the phosphorylation of MEK by the upstream kinase Raf, resulting in a shift from p-MEK to u-MEK.

To understand the mechanism of which JTP-74057 shifts p-MEK to u-MEK and prevents phosphorylation of u-MEK in cancer cells, we conducted some physicochemical analyses of MEK. As shown in Figure 4A (left and center), FCS analyses using TAMRA-conjugated JTP-74100 with the single molecule fluorescent analyzer MF20 revealed that fluorescently labeled JTP-74100 had a high binding affinity for p-MEK1, comparable to the results for JTP-74057 obtained in enzymatic analysis (Figure 2D). And, this fluorescent compound bound preferentially to u-MEK1, with a K_D_ value that was 2-fold lower than that for p-MEK. We also examined how JTP-74057 and PD0325901 displace TAMRA-conjugated JTP-74100 bound to 0.1 μM u-MEK. As shown in Figure 4A (right), the binding of TAMRA-conjugated JTP-74100 to u-MEK was displaced by JTP-74057 in a dose-dependent manner, whereas PD0325901 needed around 100-fold more concentration to replace it. These findings indicate that JTP-74057 and PD0325901 have different binding properties to u-MEK, and that the binding site of JTP-74057 in u-MEK is not exactly same to that of PD0325901.

Next, we examined the binding properties of both compounds to u-MEK using SPR. u-MEK stabilized with PD0325901 was immobilized on a sensor chip and used to assess the binding of each compound after washout of PD0325901. As shown in Figure 4B, JTP-74057 exhibited an extremely low dissociation rate constant (k_off_=1.2×10^−4^ s^−1^) to that of PD0325901 (k_off_=1.1×10^−1^ s^−1^), resulting in a very high affinity of JTP-74057 for u-MEK. The K_D_ value of JTP-74057 to u-MEK1 was 3.5×10^−10^ M, compared to 6.1×10^−8^ M for PD0325901. To further assess if JTP-74057 forms stable complex with inactive u-MEK, we examined thermal stabilities of drug-MEK complexes using a temperature-dependent CD spectropolarimeter. As shown in Figure 4C, PD0325901 marginally stabilized u-MEK, and the mid temperature of thermal unfolding (*Tm*) of u-MEK was about 50 °C in the absence or presence of PD0325901. In marked contrast, JTP-74057 significantly stabilized u-MEK and *Tm* value was shifted up to 57.5 °C.

Taken together, these findings indicate that JTP-74057 tightly binds to u-MEK in the site not to be exactly same with PD0325901, and that the mode of binding of JTP-74057 to u-MEK is of a novel type.

## DISCUSSION

In this study, we have demonstrated that the small molecule compound JTP-74057/GSK1120212/trametinib, which was discovered using cell phenotypic assays, targets MEK1/2 and binds to u-MEK with very high affinity and stability. Upon binding to MEK, JTP-74057 inhibits p-MEK activity as well as shifts the phosphorylation status of MEK from p-MEK toward u-MEK, and renders u-MEK resistant to phosphorylation. Moreover, JTP-74057 possesses the novel characteristics that the shift from p-MEK to u-MEK is rapidly induced and durably sustained. We hypothesize that the characteristics of JTP-74057 offer important molecular mechanisms for effective inhibition of the Raf–MEK–ERK signaling pathway. Figure 4D summarizes the proposed mechanism of action in this group of MEK inhibitors. As this inhibitory mechanism is unprecedented for MEK, JTP-74057 warrants consideration as a novel drug candidate targeting MEK as a therapeutic strategy for cancer patients.

As shown in the physicochemical analyses (Figure 4A, B and C), JTP-74057 possesses unique characteristics in terms of the preferential binding to, the very low dissociation rate of, and the stabilizing ability of u-MEK, which are different from those of PD0325901. These results strongly suggest that JTP-74057 extends its MEK-binding towards a novel binding site able to affect phosphorylation of Ser217/221. This phosphorylation site is thought not to be occupied by the scaffold structure of PD0325901 chemotype compounds, since PD0325901 did not increase u-MEK but accumulated p-MEK (Figure 3). Actually, it has been reported that one derivative compound of PD0325901 does not extend to the site of Ser217/221 in crystal structure analysis of MEK-compound complex [27]. Recently, inhibition of Ser217/221 phosphorylation by GSK1120212/JTP-74057 in enzyme assay and cancer cells was studied by MS analysis, and it has been shown that phosphorylation of Ser217 site but not or less of Ser221 site is preferentially inhibited by this compound [30] These previous findings and results in this study highly suggest that JTP-74057, but not PD0325901, interacts with Ser217 of u-MEK and is able to fix its inactive structure resistant of phosphorylation mediated by upper kinases such as Raf. Actually, the docking model overlaying PD compound and JTP-74057 in inactive form of MEK suggested that the acetyl amino group of JTP-74057 extends to the region of Ser217/221-containg activation loop (data not shown), though a crystal structure analysis of the JTP-74057 and u-MEK complex would be further worth to demonstrate this unique binding in detail.

This unique binding to u-MEK could explain why JTP-74057 effectively inhibits ERK phosphorylation and prolongs the unphosphorylation state of MEK. In order to understand the rapid shift of p-MEK to u-MEK by this compound (Figure 3B), however, an unrevealed feedback mechanism seems necessary to be considered, instead of known feedback mechanisms [28, 29, 31, 32]. The hyperphosphorylation of Raf by active ERK and the dephosphorylation of active ERK by dual-specific phosphatases such as DUSP6/MKP3 have been reported as two of the negative feedback mechanisms in this signaling pathway. While in the case of MEK it has not been clarified whether a negative feedback loop exists and plays a role in MEK inactivation, our data indicate that p-MEK is rapidly converted to u-MEK in the presence of JTP-74057. This may indicate that p-MEK is actively dephosphorylated by one (or both) of two possible mechanisms. The first is the existence of a MEK-specific phosphatase that constitutively dephosphorylates p-MEK, and whose action is accentuated when JTP-74057 inhibits MEK phosphorylation (i.e. the balance in the MEK phosphorylation status shifts toward dephosphorylation). The other is that p-MEK undergoes a subtle conformational change following binding of JTP-74057 that allows the known phosphatases to cross-recognize and dephosphorylate it rapidly. While p-ERK phosphatase DUSP6 and PP2A did not involve in this active conversion towards u-MEK (unpublished data), further investigation of these possibilities could reveal a novel feedback mechanism that controls activation and inactivation of MEK.

Raf kinase activity is negatively regulated by active ERK *via* hyperphosphorylation of Raf. Blockade of MEK or ERK by inhibitors disinhibits Raf and leads to p-MEK accumulation in this kinase cluster. Compounds of previously developed chemotypes, including PD0325901 and AZ6244, frequently induce accumulation of p-MEK in cancer cells and tissues, especially in B-Raf unmutated tumors [28, 29]. It was actually shown that PD0325901 induced significant accumulation of p-MEK in ACHN kidney cancer cells, which are derived from a K-Ras mutated, but B-Raf unmutated, tumor (Figure 3A). In contrast, JTP-74057 did not significantly, or slightly if any, induce accumulation of p-MEK in this B-Raf unmutated cancer cells. These findings suggest that JTP-74057, but not PD0325901, can inhibit MEK phosphorylation even when the kinase activity of Raf is disinhibited. In the case of B-Raf (V600E) mutated tumors, it has been reported that the kinase activity of Raf cannot be controlled by feedback inhibition [31, 32]. Our data showed that JTP-74057 efficiently reduced the p-MEK levels in cell lines such as HT-29 and COLO-205 that carry the V600E B-Raf mutation (Figure 3D). These findings indicate that JTP-74057 can reverse the high level of p-MEK that arises when mutated B-Raf circumvents the disabling negative feedback. This could explain why the potency of JTP-74057 in the cell phenotypic assays was higher than that in the enzymatic assays (Table 1, Figure 3A and Supplemental Figure). Taken together, in both Raf mutated and unmutated tumors, MEK inhibitors possessing the characteristics of the JTP-74057 chemotype are thought to potently and effectively inhibit Raf–MEK–ERK signaling.

Sustained inhibition of ERK phosphorylation *via* a shift to u-MEK was observed in HT-29 cells after washout of JTP-74057 from cultured cells (Fig. 3C). This prolonged shift to u-MEK is likely to arise from both the extremely low dissociation rate constant of JTP-74057 (k_off_=1.2×10^−4^ s^−1^) once bound to u-MEK and the stabilizing ability of JTP-74057 in inactive u-MEK-compound complex (Figure 4B and C). These also imply that JTP-74057 accumulates in the large u-MEK population and inhibits ERK phosphorylation via the prevention of MEK activation/phosphorylation by upstream kinases in cancer cells and tissues. In fact, it was shown that *in vivo*, JTP-74057/GSK1120212 had a long half-life in the blood and inhibited ERK phosphorylation in grafted cancer tissues after oral administration in animals [22, 30]. These findings and the present results suggest that the binding and accumulation of JTP-74057 on u-MEK leads to the sustained effects seen *in vitro* and *in vivo*. Indeed, a phase I study of GSK1120212 in cancer patients has recently shown that the t_1/2_ of orally administered trametinib is about 4 days [24]. It is likely that this ideal pharmacokinetic profile for MEK inhibition in humans is at least partly caused by the characteristics of this compound that are manifested *in vitro* as prolonged bioactivity on u-MEK.

## METHODS

### Cell lines, reagents and chemical probes

HT-29 (human colorectal adenocarcinoma cell line), ACHN (human kidney cancer cell line), HEK293T (human embryonic kidney cell line) and other types of cancer cell lines were purchased from ATCC (Manassas, VA). B-Raf (V600E), active Raf-1 (truncated), active MEK1, inactive MEK1, active MEK2, inactive MEK2, inactive MAPK2/ERK2 and bovine MBP were purchased from Upstate Technology (Waltham, MA). Other inactive MEK (K97R) was purchased from BPS Biosciences (San Diego, CA). Antibodies specific to p-MEK1/2 (Ser217/221), p-p44/42MAPK (Thr202/Tyr204), MEK1/2 and ERK1/2 were purchased from Cell Signaling Technology (Beverly, MA). The antibodies specific to p-MEK1/2 recognize Ser217-phosphorylated MEK regardless of the state of Ser221-phosphorylation. JTP-74057 chemotype compounds and PD0325901 were synthesized according to our previous report [21]. U0126 was purchased from Cell Signaling Technology. For chemical affinity chromatography and fluorescence imaging, JTP-74100 or JTP-74099 as a chemical probe was conjugated with activated CH Sepharose 4B (Amersham Biosciences, Piscataway, NJ) resins and with the succinimidyl hexanoate-linker TAMRA (5,6-linker mixture; Pierce, Rockford, IL) in bicarbonate buffer (pH 8.0) or phosphate buffer (pH 7.4). The conjugation reactions with chemical probes were monitored by HPLC, and the completion of each reaction was determined by the diminishment of the input chemical probe compounds.

### Cell culture and preparation of cell lysates

HT-29 cells were cultured in McCoy's 5a medium supplemented with 10% (v/v) fetal bovine serum and antibiotics. ACHN and HEK293T cells were maintained in Dulbecco's modified Eagle's medium supplemented with 10% (v/v) fetal bovine serum and antibiotics in a humidified CO_2_ incubator. The growth inhibitory activities of compounds toward HT-29 cells were determined by [^3^H]-TdR incorporation for the last 6 h of a 3-day culture period. To prepare the cytosolic fraction of HT-29 cells for affinity chromatography, the cells were cultured, washed with ice-cold PBS and resuspended in four volumes of a hypotonic buffer (10 mM HEPES, pH 7.9, 1.5 mM MgCl_2_, 10 mM KCl, 0.5 mM DTT) for 20 min. The lysed cells were then homogenized by 20 strokes of a loose-fitting Dounce homogenizer and centrifuged for 6 min at 4,300×*g* to separate the nuclei from the cytoplasmic fraction. The cytoplasmic fraction was ultracentrifuged for 1 h at 150,000×*g* and the supernatant was dialyzed against a buffer containing 20 mM HEPES (pH 7.9), 20% (v/v) glycerol, 0.1 M KCl, 0.2 mM EDTA, 0.5 mM PMSF and 0.5 mM DTT for 5 h at 4°C. After centrifugation for 20 min at 15,000×*g*, the supernatant was collected as the cytosolic fraction.

### Chemical probe-affinity chromatography

Activated CH Sepharose 4B resin was coupled with the chemical probes in coupling buffer (10% ethanol, 0.1 mM Na_2_CO_3_ pH 8.0) on an end-to-end rotator at room temperature for 1 h, and then blocked with 0.1 M Tris-HCl (pH 8.0) at room temperature for 1 h. The resulting resins were mixed with the HT-29 cytosolic fraction and incubated at 4°C for 1 h. The bound proteins were eluted with SDS sample buffer, heated at 95°C for 5 min and separated by SDS-PAGE. The gels were developed by silver staining. For competition analyses using compounds from the same chemotype and known MEK inhibitors, HT-29 cytosolic fractions were incubated with or without the various compounds on ice for 30 min, followed by the pull-down assay described above.

### Enzymatic kinase assays

MEK–ERK kinase assays were carried out in 20 mM MOPS (pH 7.2), 25 mM β-glycerophosphate, 5 mM EGTA, 1 mM DTT, 1 mM Na_3_Vo_4_, 15 mM MgCl_2_, 10 μM ATP, 1 μCi [^32^P]ATP, 10 nM p-MEK (p-MEK1 or p-MEK-2) and 300 nM u-ERK2. The Raf–MEK–ERK kinase assays were carried out in 50 mM Tris-HCl (pH 7.5), 0.1 mM EGTA, 0.03% (v/v) Brij35, 0.1% (v/v) 2-mercaptoethanol, 1.5 mM MgCl_2_, 10 μM ATP, 0.4 μCi [^32^P]ATP, 0.6 nM active Raf (B-Raf or c-Raf), 10 nM u-MEK (u-MEK1 or u-MEK 2) and 300 nM u-ERK2 [26]. After incubation of the components (except ATP) for 15 min at 30°C, the assays were initiated by adding ATP. After 20 min, the samples were denatured with Laemmli SDS sample buffer and subjected to PAGE, and the radioactivity was analyzed using a phosphorimager (BAS 2000; Fujifilm, Tokyo, Japan). The purity of u-MEK was measured by western blotting using antibodies specific to p-MEK, and it was confirmed that there was no detectable p-MEK in 500 pg of total u-MEK (data not shown). Raf–MBP kinase assays were carried out in an identical manner, except that the concentrations of B-Raf and c-Raf were increased by 100- and 10-fold respectively. For kinetic analyses of MEK inhibition, double-reciprocal plot analyses were conducted in the presence of various concentrations of the compounds. The Ki (MEK *vs*. compound) and Ki’ (MEK/ATP *vs*. compound) values were determined from the plots as previously described [33].

### Western blot analysis

Cultured cells were treated with various compounds and then lysed in lysis buffer (50 mM Tris-HCl pH 7.4, 150 mM NaCl, 1 mM EGTA, 1 mM Na_3_Vo_4_, 1% Triton X-100 and EDTA-free protease inhibitor cocktail). The cell lysates were collected and centrifuged, and the supernatants were subjected to SDS-PAGE. After the electrophoresis, the proteins were transferred onto PVDF membranes at 1 mA/cm^2^ for 1 h. The membranes were incubated in blocking solution (Blockace; Nacalai Tesque, Kyoto, Japan) and washed with PBS containing 0.1% Tween 20 (PBS/T). The membranes were then incubated with the primary antibody, washed three times with PBS/T and incubated with the secondary antibody. After washing with PBS/T, the membranes were incubated with ECL-Plus or ECL-Advanced western blot reagents (GE Healthcare, Little Chalfont, Buckinghamshire, UK) for 5 min, exposed to ECL Hyperfilm and visualized using a LAS3000 imager (Fujifilm).

### Fluorescence microscopy

The pcDNA3.1(+) (Clontech, Palo Alto, CA) or pCMV6/human MEK2 (OriGene Technologies, Rockville, MD) vectors were transfected into HEK293T cells using GeneJammer (Stratagene, La Jolla, CA). The transfected cells were plated in 6-well culture plates at 3×10^5^ cells/well and treated with DMSO or 1 μM TAMRA-conjugated JTP-74100 overnight. After replacing the medium with normal medium to remove excess fluorescent probe, the cells were fixed with 4% paraformaldehyde in PBS supplemented with 10 μM Höechst 33258 at room temperature for 20 min and visualized using an A1 fluorescence microscope (Nikon, Tokyo, Japan).

### Fluorescence correlation spectroscopy (FCS)

FCS analysis was performed with a single molecule fluorescence analyzer (MF20; Olympus, Tokyo, Japan). Several concentrations of p-MEK1 and u-MEK1 were incubated with 1.5 nM of TAMRA-conjugated JTP-74100 for 30 min at 25°C in binding buffer (20 mM Hepes pH 7.4, 150 mM NaCl, 0.1% Tween 20). The samples were added to a 384-well glass-bottomed microplate, and FCS measurements were performed in a sample volume of 30 μl with excitation at 543 nm (He/Ne laser) to detect TAMRA-derived red fluorescence. All experiments were performed under identical conditions with a data acquisition time of 5 s per measurement. Measurements were repeated five times per sample. The equilibrium dissociation constant (K_D_) value was calculated by Origin™ software (OriginLab Corporation, Northampton, MA) according to a previously described method [34].

### Surface plasmon resonance (SPR)

SPR measurements were performed using a Biacore S51 (GE Healthcare Life Sciences, Piscataway, NJ). For stabilization, u-MEK1 was diluted to 25 μg/ml in 10 mM sodium citrate (pH 6.0), 1% DMSO and 100 μM PD0325901 shortly before immobilization. The stabilized enzyme was then immobilized on a CM5 sensor chip by amide coupling chemistry at 25°C using HBS-P+ (GE Healthcare Life Sciences) as a running buffer. The surface of the sensor chip was activated with a mixture of 0.1 M *N*-hydroxysuccinimide and 0.4 M *N*-ethyl-*N’*-(3-dimethylaminopropyl)carbodiimide for 10 min. Subsequently, u-MEK1 was injected for 7 min. Any remaining activated ester groups were blocked with 1 M ethanolamine (pH 8.5) for 10 min. The immobilization levels typically ranged from 6,600–10,000 resonance units. Interaction studies were conducted at 25°C in a running buffer containing 50 mM Tris-HCl (pH 7.5), 150 mM NaCl, 1.5 mM MgCl_2_, 1 mM DTT, 0.005% Tween 20 and 1% DMSO. Compounds were diluted directly into the running buffer. A typical analysis cycle consisted of a 60-s sample injection (90 μl/min, association phase) followed by buffer flow for 180 s (dissociation phase). Analyses of the resulting sensorgrams were performed using Biacore S51 Evaluation software (GE Healthcare Life Sciences). Kinetic parameters were obtained by global fitting for PD0325901 or local fitting for JTP-74057 to a 1:1 binding model with mass transfer.

### Temperature-dependent circular dichroism (CD)

The CD experiments were performed Jasco J720 spectropolarimeter (Tokyo, Japan) with a piezoelectric temperature controller, according to the previous report [35] with slight modification. In brief, ellipticity was monitored at 229 nm as a function of temperature with 1 mm path length cell, and the scan rate was 1 °C per min. Inactive u-MEK (K97R) protein was diluted to 3 μM with 25 mM HEPES at pH 7.5, 150 mM NaCl, 10% glycerol, 1 mM DDT, and 0.1% DMSO. JTP-74057 and PD0325901 were tested at 10 μM concentrations. The representative temperature-dependent ellipticity profiles were depicted, and the midpoints of the protein unfolding transition (*Tm*) were calculated as the average from two separate experiments, according to the calculation equation described in the previous report [36].

## Supplementary Table and Figures


