# Supplementary material for: Identification and Characterization of a Novel Chemotype MEK Inhibitor Able to Alter the Phosphorylation State of MEK1/2
Source: Oncotarget. 2012 Dec 1;3(12):1533–45. doi: 10.18632/oncotarget.747 (PMC3681492; doi:10.18632/oncotarget.747)

Identification and Characterization of a Novel Chemotype MEK Inhibitor Able to Alter the Phosphorylation State of MEK1/2 – Yoshida et al

Synthesis and NMR Data of Chemical probes JTP-74100, JTP-74099 and Other Compounds. The synthetic procedure and characterization data for JTP-74057 have been reported (Abe H. et al; 2011). The syntheses of other JTP-compounds were carried out according to the general synthetic method as described in the same reference. The characterization data of compounds in **Figure 1** are as follows, except for JTP-74057 and PD0325901.

JTP-70945.

N-{3-[3-Cyclopropyl-5-(2-fluoro-4-iodophenylamino)-8-methyl-2,4,7-trioxo-3,4,7,8-tetrahydro-2*H*-pyrido[2,3-*d*]pyrimidin-1-yl]phenyl}methane sulfonamide. ^{1}H NMR (300 MHz, DMSO-d_6) δ 0.74 (m, 2H), 1.03 (m, 2H), 2.65 (s, 4H), 3.02 (s, 3H), 5.38 (s, 1H), 7.13 (d, *J* = 8.6 Hz, 1H), 7.20–7.40 (m, 3H), 7.46 (t, *J* = 8.1 Hz, 1H), 7.63 (d, *J* = 9.3 Hz, 1H), 7.81 (d, *J* = 10.1 Hz, 1H), 10.00 (s, 1H), 10.54 (s, 1H); MS (ESI) *m*/*z* 638 $(M+H)^+$.

JTP-65634.

5-(2-Methoxyphenylamino)-8-methyl-1,3-diphenyl-1*H*,8*H*-pyrido[2,3-*d*]pyrimidine-2,4,7-trione. ^{1}H NMR (400 MHz, $CDCl_3$) δ 2.89 (s, 3H), 3.83 (s, 3H), 5.83 (s, 1H), 6.95 (t, *J* = 8.1 Hz, 2H), 7.16 (td, *J* = 7.2, 1.6 Hz, 1H), 7.28–7.32 (m, 2H), 7.35–7.54 (m, 9H), 10.21 (s, 1H); MS (ESI) *m*/*z* 467 $(M+H)^+$.

JTP-74100.

3-Amino-*N*-{3-[3-cyclopropyl-5-(2-fluoro-4-iodophenylamino)-6,8-dimethyl-2,4,7-trioxo-3,4,6,7-tetrahydro-2*H*-pyrido[4,3-*d*]pyrimidin-1-yl]-phenyl}propionamide hydrochloride. ^{1}H NMR (300 MHz, DMSO-d_6) δ 0.66 (m, 2H), 0.95 (m, 2H), 1.26 (s, 3H), 2.62 (m, 1H), 2.73 (t, *J* = 6.6 Hz, 2H), 3.08 (s, 3H), 3.09 (m, 2H), 6.92 (t, *J* = 8.5 Hz, 1H), 7.06 (d, *J* = 7.5 Hz, 1H), 7.39 (t, *J* = 7.9 Hz, 1H), 7.53–7.64 (m, 2H), 7.67 (s, 1H), 7.78–7.88 (m, 4H), 10.37 (s, 1H), 11.07 (s, 1H); MS (ESI) *m*/*z* 645 $(M+H)^+$.

JTP-74099.

3-Amino-*N*-{3-[3-cyclopropyl-5-(2-fluorophenylamino)-6,8-dimethyl-2,4,7-trioxo-3,4,6,7-tetrahydro-2*H*-pyrido[4,3-*d*]pyrimidin-1-yl]-phenyl}propionamide hydrochloride. ^{1}H NMR (300 MHz, DMSO-d_6) δ 0.68 (m, 2H), 0.96 (m, 2H), 1.26 (s, 3H), 2.63 (m, 1H), 2.73 (t, *J* = 6.4 Hz, 2H), 3.05 (s, 3H), 3.07 (m, 2H), 7.06 (d, *J* = 9.0 Hz, 1H), 7.11–7.32 (m, 3H), 7.32–7.43 (m, 2H), 7.61 (d, *J* = 7.9 Hz, 1H), 7.67 (s, 1H), 7.83 (brs, 3H), 10.38 (s, 1H), 11.23 (s, 1H); MS (ESI) *m*/*z* 519 $(M+H)^+$.

Peptide Sequences Identified in 46 kDa Proteins by LC-MS/MS Analysis.

Proteins of 46 kDa band (**Figure 2A**) were identified as MEK1 and MEK2 by LC-MS/MS and Mascot search. In brief, the individual protein bands in SDS-PAGE gels were excised and subjected to in-gel digestion with trypsin. The digested peptides were desalted and analyzed with a nanoLC-MS/MS system with a Q-ToF mass spectrometer (Waters Micromass). The NCBInr database was searched using the Mascot search engine (Matrix Science) to identify homologous proteins As shown in the **Supplemental Table**, the coverage of MEK1 and MEK2 by the identified peptides was 24% and 12%, respectively, without any significant homologies to other human proteins.

Supplemental Table: Identified Peptide Sequences of 46 kDa Proteins.

ACCESSION /protein name	Start – End /score	Obsrved m/z	Mr(expt)	Mr(calc)	Sequence
	4–36 / 86	819.95	3275.77	3275.70	KKPTPIQLNPAPDGSAV –NGTSSAETNLFALQK
gi 5579478	60–70 /30	432.22	1293.63	1293.61	VGELKDDDFEK
/mitogen-	60–70 /62	647.83	1293.65	1293.61	VGELKDDDFEK
activated	71–84 /79	674.83	1346.75	1346.72	ISELGAGNGGVVFK
protein kinase	85–96 /16	641.40	1280.79	1280.70	VSHKPSGLVMAR
kinase 1	161–168 /40	449.28	896.55	896.63	PEQILGK
	190–201 /26	671.40	1340.79	1340.74	DVKPSNILVNSR
	261–269 /30	499.28	996.56	996.53	YPIPPPFAK
hi 13489054	75–86 /67	651.38	1300.74	1300.70	ISELGAGNGGVVTK
/mitogen-	194–205 /26	671.40	1340.79	1340.74	DVKPSNILVNSR
activated	265–273 /30	499.28	996.55	996.53	YPIPPPDAK
protein kinase	333–348 /20	926.49	1850.97	1850.92	LPNGVFTPDFQEFVNK
kinase 2	333–348 /29	926.50	1850.99	1850.92	LPNGVFTPDFQEFVNK

Supplemental Figure: Alterations of downstream signaling molecules in cancer cells by JTP-74057 and PD0325901. (A, B) HT-29 cells (A) and ACHN cells (B) were treated with the indicated concentrations of each compound for 24 h, same as the experiment of **Figure 3A**. And the cell lysates were analyzed by western blotting using antibodies specific to c-Myc (Upstate Technology), cyclin D1, $p15^{INK4b}$, $p27^{Kip1}$ and α-tubulin (Santa Cruz Biotechnology, Santa Cruz, CA). The accumulation of $p15^{INK4b}$ and $p27^{KIP1}$, and the decrease of c-Myc and Cyclin D1 were observed at 1 nM of JTP-74057 and 10 nM of PD0325901, respectively.

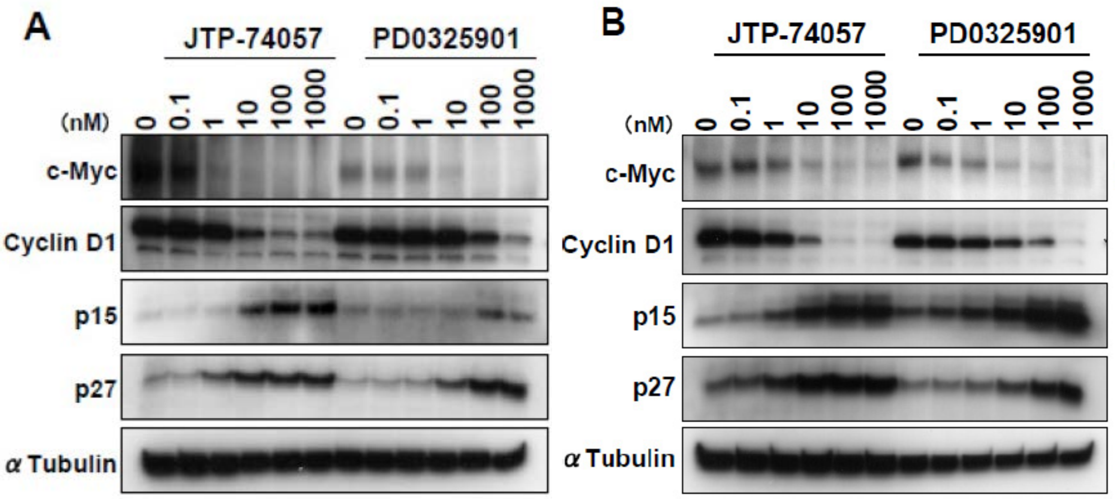

Supplement: Supplementary file 1 [file oncotarget-03-1533-s001.pdf]
